# A non-enzymatic electrochemical hydrogen peroxide sensor based on copper oxide nanostructures

**DOI:** 10.3762/bjnano.13.35

**Published:** 2022-05-03

**Authors:** Irena Mihailova, Vjaceslavs Gerbreders, Marina Krasovska, Eriks Sledevskis, Valdis Mizers, Andrejs Bulanovs, Andrejs Ogurcovs

**Affiliations:** 1 G. Liberts' Innovative Microscopy Centre, Department of Technology, Institute of Life Sciences and Technology, Daugavpils University, Parades Street 1, Daugavpils, LV-5401, Latvia; 2 Institute of Solid State Physics, University of Latvia, Kengaraga street 8, Riga, LV-1063, Latvia

**Keywords:** copper oxide, electrochemical sensor, hydrogen peroxide, nanostructures

## Abstract

This article describes the synthesis of nanostructured copper oxide on copper wires and its application for the detection of hydrogen peroxide. Copper oxide petal nanostructures were obtained by a one-step hydrothermal oxidation method. The resulting coating is uniform and dense and shows good adhesion to the wire surface. Structure, surface, and composition of the obtained samples were studied using field-emission scanning electron microscopy along with energy-dispersive spectroscopy and X-ray diffractometry. The resulting nanostructured samples were used for electrochemical determination of the H_2_O_2_ content in a 0.1 M NaOH buffer solution using cyclic voltammetry, differential pulse voltammetry, and *i*–*t* measurements. A good linear relationship between the peak current and the concentration of H_2_O_2_ in the range from 10 to 1800 μM was obtained. The sensitivity of the obtained CuO electrode is 439.19 μA·mM^−1^. The calculated limit of detection is 1.34 μM, assuming a signal-to-noise ratio of 3. The investigation of the system for sensitivity to interference showed that the most common interfering substances, that is, ascorbic acid, uric acid, dopamine, NaCl, glucose, and acetaminophen, do not affect the electrochemical response. The real milk sample test showed a high recovery rate (more than 95%). According to the obtained results, this sensor is suitable for practical use for the qualitative detection of H_2_O_2_ in real samples, as well as for the quantitative determination of its concentration.

## Introduction

Hydrogen peroxide, a strong oxidant and an essential intermediate product in many biomedical reactions, has recently attracted widespread research interest. In high concentrations it can cause serious harm to human health and the environment, despite the fact that, in living organisms, H_2_O_2_ is a by-product of metabolism for a wide range of biological and chemical processes, occurring under the influence of external stimuli and intracellular processes [1–2]. Disruption of the natural regulation process and increasing concentration of H_2_O_2_ in the blood can cause severe diseases such as Alzheimer's and Parkinson's [3], premature aging of cells [4], death of nerve cells [3,5–6], loss of brain mass [7], and cancer [8–11]. For this reason, targeted monitoring of the concentration of H_2_O_2_ in body fluids can be used in the diagnosis of these diseases [12–15]. Rapid and accurate determination and control of H_2_O_2_ concentration is an important task in many other areas, including pharmaceuticals [16–18], environmental protection [19], and industrial areas (especially food production) [20–25].

Measurement techniques including fluorescence [26–27], luminescence [28], spectrometry [29–30], and electrochemistry [31–33] are widely used for H_2_O_2_ determination. Currently, the electrochemical method is most widely used due to its simplicity, selectivity, and low detection limit. Modified (with enzymes) and unmodified electrodes are used as working electrodes. In the case of modified electrodes, the surface is functionalized by redox-active enzymes (the most popular being horseradish peroxidase) [34–36], and detection is carried out through physicochemical processes of interaction between H_2_O_2_ and the enzyme. This type of sensor has high catalytic activity, sensitivity, and selectivity. However, enzyme sensors have a significant disadvantage, namely enzyme instability. Due to the nature of enzymes, they can be easily damaged thermally and chemically during production, transportation, and use of electrodes. In addition, enzymes are quite expensive, which significantly increases the production cost and total price of this type of sensor. Recently, research has focused on the development of non-enzymatic electrochemical sensors for the detection of H_2_O_2_ [37–39]. In this type of sensor, H_2_O_2_ interacts with the electrode material directly. Certain catalytic processes occurring between H_2_O_2_ and the electrode material provide an unambiguous electrochemical response and, as a consequence, the selectivity of the sensor. This type of sensor is characterized by good reproducibility of measurement, low production cost, fast response, high sensitivity and selectivity, and chemical and mechanical stability in aggressive environments [40–46]. Nanostructured materials are widely used as the working surface of the electrode [47–49]. The most common are transition metal nanoparticles [33,37,50–54], carbon nanotubes [8], metal oxides [55–64], graphene [32–33], and ordered mesoporous carbon [38,65–66]. Compared to bulk materials, nanostructures have higher catalytic activity and a significantly increased surface area-to-volume ratio, which makes it possible to significantly increase both sensitivity of the sensor and rate of detection of H_2_O_2_. Among the nanostructured materials used, the most promising candidate is copper oxide (CuO) [56,67–71]. It has selectivity for the determination of H_2_O_2_, high catalytic activity, and a variety of morphologies (e.g., nanoneedles, nanoplates, and nanorods). Various techniques have been used in the preparation of nanostructured epitaxial CuO coatings, such as thermal oxidation of copper electrodes in an oxygen atmosphere [72–73], hydrothermal chemical oxidation of copper surfaces [56], and hydrothermal synthesis using various precursors containing copper ions [74–75]. Copper oxide nanostructures can also be obtained as a powder and then applied to electrodes by dip- or drop-coating techniques, using a porous substrate or binder polymers [69,76–77]. However, despite the widespread use and simplicity of this method of electrode preparation, it has a number of significant disadvantages. First, there is the problem of homogenization of the nanostructured suspension in solution. Second, nanostructures are distributed randomly during the process of deposition, which can affect the electrochemical activity of the electrode and reduce the repeatability of the experiment. Third, the obtained coatings are characterized by their low adhesion and poor mechanical stability, and can, thus, be easily damaged during production, storage, and measurement. These disadvantages can be avoided by using an in situ growth process of CuO nanostructures directly on a copper substrate, in the presence of certain surfactants or additives. This method makes it possible to obtain nanostructures with a large active surface area, which ensures efficient electron charge transfer between CuO nanostructures and the copper substrate due to the formation of high-density, single-crystal nanopetals. Nanostructures are produced in one step, and can be directly used as sensor electrodes without additional treatments such as surface modification or enzyme immobilization. This article describes the process of obtaining wire electrodes with nanostructured CuO coatings by a one-step chemical hydrothermal oxidation method and their application in electrochemical measurements for the detection of H_2_O_2_. The article proves the higher efficiency of nanostructured electrodes compared to electrodes with less developed surface. The article shows the influence of the time of hydrothermal synthesis on the morphology of nanostructures and, as a result, the change in the sensitivity of the sensor. The most important electrochemical measurements were carried out to determine H_2_O_2_ concentration in aqueous solutions using the obtained sensor. It is shown that the obtained non-enzymatic sensor has high sensitivity and selectivity toward H_2_O_2_. Experiments were also carried out to detect H_2_O_2_ in real milk and mouthwash samples.

## Materials and Methods

### Materials

Ammonium persulfate ((NH_4_)_2_S_2_O_8_, CAS number: 7727-54-0), sodium hydroxide (NaOH, CAS number: 1310-73-2), and hydrogen peroxide solution (H_2_O_2_, 30%, CAS number: 7722-84-1) were purchased from Merck. Ascorbic acid (C_6_H_8_O_6_, CAS number: 50-81-7), uric acid (C_5_H_4_N_4_O_3_, CAS number: 69-93-2), dopamine hydrochloride ((HO)_2_C_6_H_3_CH_2_CH_2_NH_2_HCl, CAS number: 62-31-7), glucose (C_6_H_12_O_6_, CAS number: 50-99-7), acetaminophen (CH_3_CONHC_6_H_4_OH, CAS number: 103-90-2), and sodium chloride (NaCl, CAS number: 7647-14-5) were purchased from Sigma-Aldrich. All reagents were ≥99.8% pure. Copper wire of 2 mm thickness (99.9% purity) was purchased from Sigma-Aldrich. Ag/AgCl wire was purchased from A-M Systems, USA. Printed circuit boards (PCBs) with ENIG (Electroless Nickel Immersion Gold) surface finish were purchased from Multi-CB (Germany). Distilled water was obtained in the laboratory.

### CuO layer synthesis on copper wires

A smooth film coating of copper oxide was obtained by annealing the copper wire in an oxygen atmosphere. Before annealing, the copper wire was washed several times with water and ethanol to clean the surface of possible contamination. The wire was then fixed in a metal holder and placed in a Linn High Therm (Germany) furnace, where it was gradually heated to 500 °C and held at this temperature for 30 min. Then, the oven was turned off and left to cool naturally. The result was a wire with a uniform black coating.

Nanostructured samples were obtained by a one-step chemical hydrothermal oxidation. For this, copper wire was rinsed with water and ethanol in order to clean the surface of possible contamination. To prepare the working solution, 10 mL of a 10 M NaOH solution, 5 mL of a 1 M (NH_4_)_2_S_2_O_8_ solution and 26 mL of H_2_O were combined. The wire samples were immersed in the resulting solution and then poured into a heat-resistant glass beaker with a lid. The beaker was placed in an oven preheated to 90 °C for 3 h, and then left to cool naturally. The obtained samples, covered with a nanostructured oxide layer, were repeatedly washed with distilled water in order to get rid of residual reagents, and then dried in an oven at 90 °C for 3 h in order to remove moisture.

To compare the dependence of the sensitivity of nanostructured samples on their morphology, samples were obtained after 1 and 6 h of synthesis time.

The morphology of the surface of the nanostructured CuO samples was studied via field-emission scanning electron microscopy (FESEM, Tescan MAIA 3). The chemical composition analysis was performed via energy-dispersive spectroscopy (EDS, Inca Synergy) integrated into the FESEM system.

The crystalline structure of the samples was defined using an X-ray diffractometer (RIGAKU Smart Lab, Cu Kα [λ = 1.543 Å]) using parallel beam scanning geometry and an additional Ge(220) × 2 bounce monochromator.

### Electrochemical measurements

The obtained wire samples were cut into 2 cm long pieces, and at one end were stripped to pure copper over 5 mm length to provide electrical contact with the equipment. The measurements were carried out using an electrochemical station (Zanher, Germany), supplemented by a custom-made electrochemical cell (for more details about its structure, see our publication [71]). During the measurement, a three-electrode cell was used, using oxide-coated copper wire as a working electrode, 0.4 mm diameter Ag/AgCl wire as a reference electrode, and a 6 × 6 mm PCB electrode with ENIG surface finish as a counter electrode.

Cyclic voltammetry (CV) was carried out in the range from −0.8 to 0.1 V vs Ag/AgCl, with *U*_start_ = 0 V vs Ag/AgCl and a scan rate of 100 mV/s. As buffer solution, 0.1 M NaOH (pH 12.7) was used. For the determination of H_2_O_2_, 0.1, 0.25, 0.5, 0.65, 0.85, 1, and 5 mM concentrations were used. Measurements were carried out five times for each of the indicated concentrations, and the curves in the following sections show the averaged data from all measurements. To determine the optimal scanning parameters that provide the maximum sensitivity of the sensor, the dependence of the electrochemical response on the pH of the buffer solution and on the scanning speed was studied.

Impedance spectroscopy was carried out in the frequency range from 1 Hz to 100 kHz at an applied signal voltage of about 0.3V.

### Differential pulse voltammetry

Before the measurement, the samples were maintained for 30 s at *U* = −0.8 V vs Ag/AgCl. The measurements were carried out using the following parameters: voltage range from −0.8 V to 0.1 V vs Ag/AgCl, pulse amplitude = 50 mV, pulse step = 3 mV, pulse width = 200 ms, and pulse frequency = 2 Hz. As buffer solution, 0.1 M NaOH was used. For the determination of H_2_O_2_, 0.033, 0.066, 0.1, 0.17, 0.25, 0.37, and 0.5 mM concentrations were used. The measurements were carried out five times for each of the indicated concentrations, and the curves in the following sections show the averaged data from all measurements.

To determine the scanning parameters that provide the maximum sensitivity of the sensor, the dependence of the differential pulse voltammetry (DPV) response on the pH of the buffer solution and on the pulse frequency was studied.

### Current response study

For the current response study (*i*–*t* measurement), a constant voltage *U* = −0.7 V vs Ag/AgCl was applied to the cell and the current was measured. 0.1 M NaOH was used as buffer solution. The measurement was started at 0 µM concentration, and after 600 s (time required for stabilization) the first 10 µM portion of H_2_O_2_ was added. Subsequent portions were added every 30 s with the following steps: 10 µM for the concentration range of 0–100 µM, 20 µM for the concentration range of 120–300 µM, 50 µM for the concentration range of 350–800 µM, and 100 µM for the concentration range of 900–1800 µM. The measurement was carried out with constant stirring using a magnetic stirrer.

### Interference study

A constant voltage *U* = −0.7 V vs Ag/AgCl was applied to the cell and the current was measured. As buffer solution 0.1 M NaOH was used. The experiment was started at 0 µM concentration of H_2_O_2_, then every 60 s either H_2_O_2_ or an interfering substance at a concentration of 100 µM was added to the solution, in the following order: H_2_O_2_, ascorbic acid, uric acid, dopamine, NaCl, glucose, and acetaminophen. Then, the whole cycle was repeated two times. The measurement was carried out with constant stirring using a magnetic stirrer.

### Real sample study

To demonstrate the possibility of practical application of the obtained nanostructured electrodes for the analysis of real samples, samples of ultrahigh-temperature processed (UHT) milk were investigated. H_2_O_2_ is present in milk samples either as a result of enzymatic activity or as an antibacterial agent [20–22]. For the experiment, we used 3.2% fat milk and Listerine antiseptic mouthwash from a local supermarket. To reduce the sample matrix effect, the samples were diluted in a 1:2 ratio with 0.1 M NaOH buffer solution. The resulting solution was maintained at pH 12.7. The amperometric response method was used for the analysis with *U* = −0.7 V vs Ag/AgCl.

## Results and Discussion

### CuO structure

**Figure 1:**
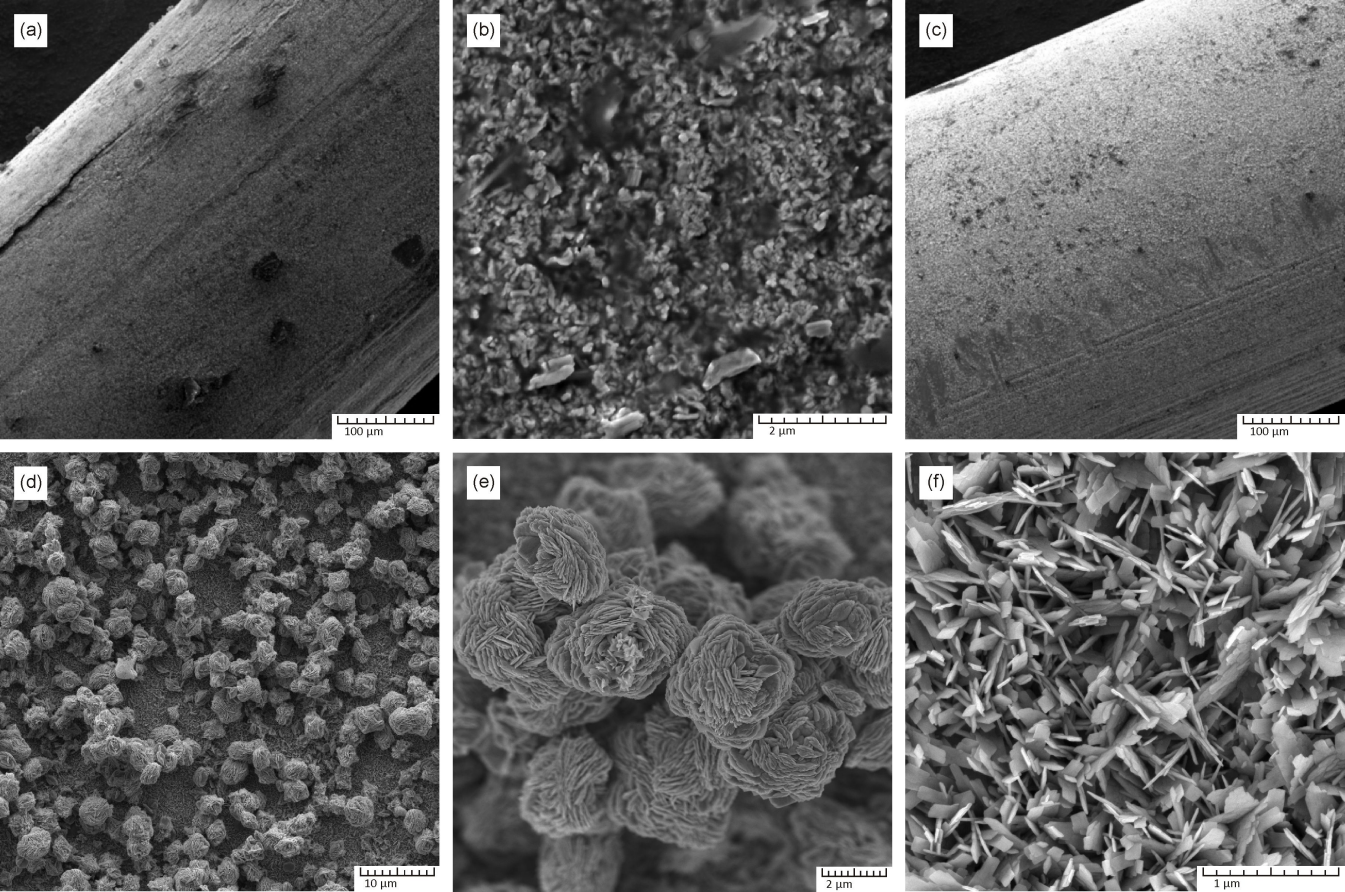
SEM images of copper oxide samples. (a, b) General view and morphology of a CuO film obtained by thermal oxidation on a copper wire; (c, d) general view of a copper wire with CuO layer obtained by chemical hydrothermal oxidation; (e) 3D flower-like nanostructured formations on the surface of the main CuO layer (f).

The morphology of CuO is shown in Figure 1. The SEM image (Figure 1a,b) shows the surface morphology of a thermally obtained copper oxide film. The resulting film is a homogeneous, polycrystalline oxide layer consisting of grains of arbitrary shape. In practice, this layer exhibits poor adhesion to the surface and can be easily damaged mechanically during post-processing.

Figure 1c–f shows the morphology of the copper oxide layer obtained by chemical hydrothermal oxidation. The resulting coating is characterized by a high degree of uniformity, good adhesion to the copper surface and stability during post-processing. The resulting coating consists of a dense uniform layer of CuO petals several nanometres thick (Figure 1f). The surface of the main layer is covered with chaotically distributed, micrometre-sized 3D flower-like formations assembled from individual petals (Figure 1d,e).

EDS microanalysis showed that the samples consist of Cu (58.96 atom %) and O (41.04 atom %), which confirms the high chemical purity of the samples obtained and the absence of foreign impurities.

**Figure 2:**
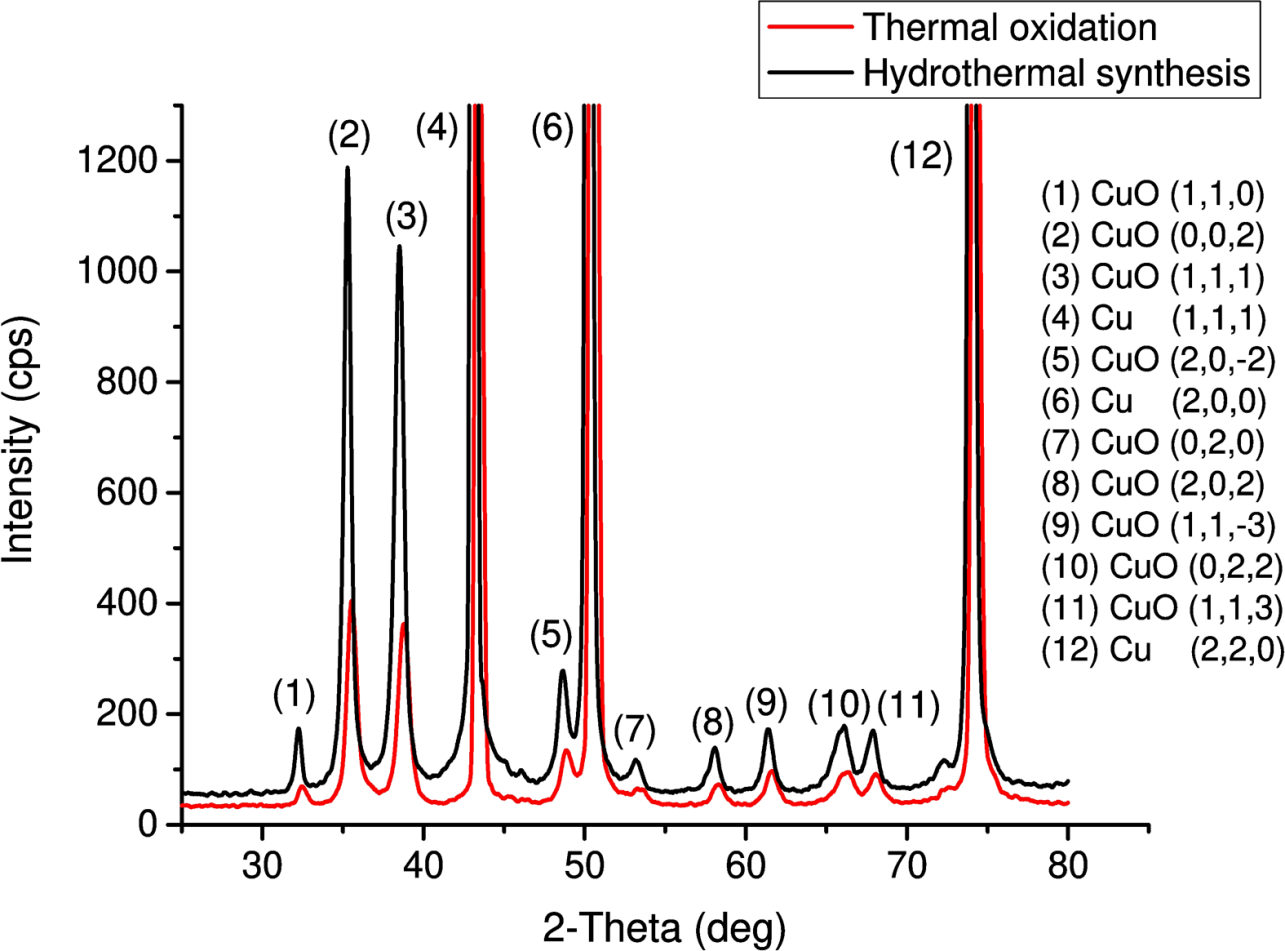
XRD pattern of CuO films. The red diffractogram corresponds to the sample obtained by thermal oxidation and the black diffractogram corresponds to the sample obtained by chemical hydrothermal oxidation.

Figure 2 shows the XRD analysis results. The diffractogram shows only peaks corresponding to CuO and pure Cu (substrate peaks). Extraneous phases and inclusions were not detected. A low amorphous background indicates a high degree of crystallinity of the obtained samples. The X-ray diffraction pattern shows a large number of crystallographic planes corresponding to the CuO (tenorite) lattice; however, the dominant orientation corresponds to the direction perpendicular to the (002) and (111) planes.

The growth process of nanostructures can be explained as per the following reactions:


[1] $$\begin{array}{l} \text{Cu}+2\text{NaOH}+{\left({\text{NH}}_{4}\right)}_{2}{\text{S}}_{2}{\text{O}}_{8}\\ \to \text{Cu}{\left(\text{OH}\right)}_{2}+{\text{Na}}_{2}{\text{SO}}_{4}+{\left({\text{NH}}_{4}\right)}_{2}{\text{SO}}_{4} \end{array}$$

[2] $$\text{Cu}{\left(\text{OH}\right)}_{2}+2{\text{OH}}^{-}\to {\left[\text{Cu}{\left(\text{OH}\right)}_{4}\right]}^{2-}$$

[3] $${\left[\text{Cu}{\left(\text{OH}\right)}_{4}\right]}^{2-}\to \text{CuO}+2{\text{OH}}^{-}+{\text{H}}_{2}\text{O}$$

When NaOH is added to the precursor solution containing (NH_4_)_2_S_2_O_8_, Cu^2+^ ions are released from Cu into solution, where they interact with the reagents according to Equation 1. Reference [56] mentions that at NaOH concentrations below 5 M a thin Cu(OH)_2_ film is instantly formed on the copper surface. This film serves as a protective layer and blocks all further reactions, including crystal growth. The same processes are observed in the case when the reaction proceeds at relatively low temperatures, which explains why it is impossible to obtain the developed nanostructured CuO surface at room temperature. However, after increasing the concentration of NaOH to 10–15 M, the dissolution–secondary precipitation mechanism takes effect: Cu(OH)_2_ reacts with OH^−^ ions to form the complex ion [Cu(OH)_4_]^2−^ (Equation 2). These complex ions decompose to CuO with a loss of two hydroxy ions and one water molecule (Equation 3). As a result of this process, a large number of nuclei are generated and captured by the surface. The growth of organized, evenly distributed petal-shaped nanostructures over the entire surface of the copper wire is observed.

This process is similar to the conventional hydrothermal growth of most metal oxides described in previous studies [74,78–79]; however, this work has a fundamental difference: Cu-containing salts are not used in the synthesis process. The copper wire itself acts as the precursor of Cu ions as well as a substrate for the nanostructure growth. In this case there is no need to use an additional seed layer of CuO [74], which greatly simplifies the electrode manufacturing process and improves the adhesion of the nanostructured layer to Cu.

The spherical shape of the obtained flower-like nanostructures indicates that their nucleation centre is not located in the plane of the substrate. The formation of spherical structures can be explained as follows: the presence of a large number of OH^−^ ions makes it possible to generate a large number of nucleation centres in solution in a short time. The particles begin to agglomerate in order to minimize the total surface energy, forming spherical seeds, which, according to the mechanism of dissolution–secondary precipitation [78,80], overgrow with CuO petals, thereby forming 3D structures in solution. Then, under the influence of gravity, these structures gradually descend to the substrate, where they are captured by the surface and immobilized.

### Electrochemical measurements

**Figure 3:**
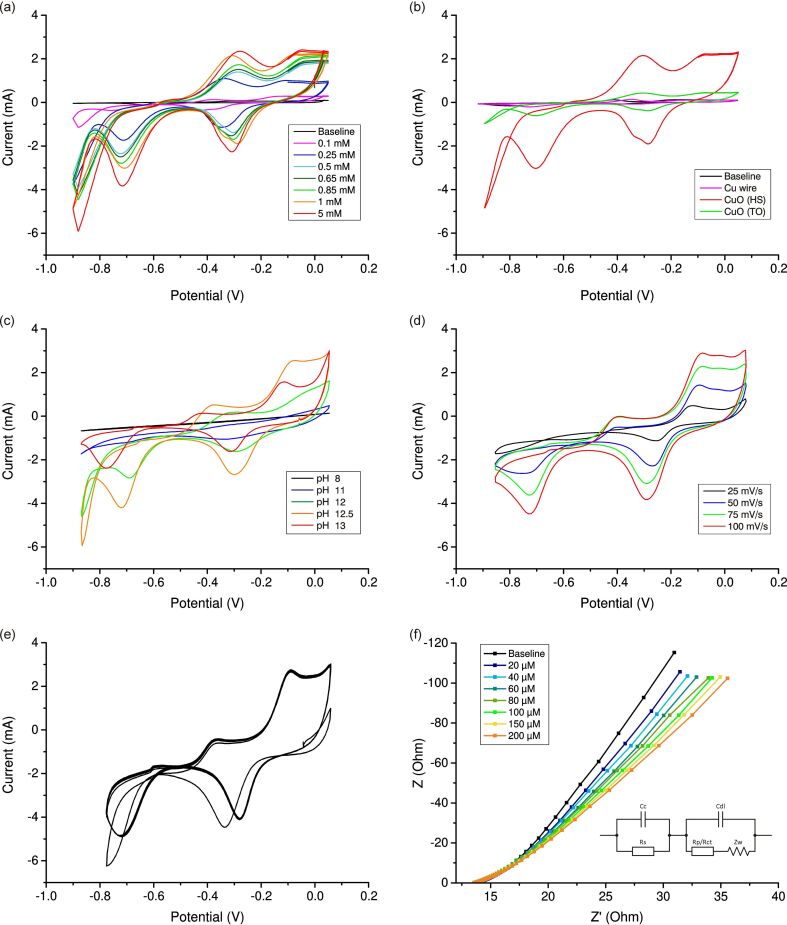
(a) CV results for a nanostructured CuO film in 0.1 M NaOH buffer solution (pH 12.7) and in solutions containing 0.1–5 mM H_2_O_2_. (b) Comparison of CV results for a pure Cu wire, a CuO film obtained via thermal oxidation (TO), and a nanostructured CuO film obtained by hydrothermal synthesis (HS). Measurements were carried out in 0.1 M NaOH solution containing 5 mM H_2_O_2_. (c) Comparison of CV curves obtained at different pH values of buffer solution containing 5 mM H_2_O_2_. (d) Comparison of CV curves obtained at different scan speeds. Measurements were carried out in 0.1 M NaOH solution containing 5 mM H_2_O_2_. (e) Electrode stability study over multiple CV cycles (*n* = 10). Measurements were carried out in 0.1 M NaOH solution containing 5 mM H_2_O_2_. (d) EIS analysis (frequency range from 1 Hz to 100 kHz at an applied signal voltage of about 0.3 V). Measurements were carried out in 0.1 M NaOH solution containing 0–200 μM H_2_O_2_.

Figure 3 shows the CV results for CuO in the solution containing 0.1 M NaOH and H_2_O_2_ at various concentrations. The curve shows a pair of oxidation peaks corresponding to Cu^0^/Cu^+^ and Cu^+^/Cu^2+^ transitions, as well as a pair of reduction peaks corresponding to Cu^2+^/Cu^+^ and Cu^+^/CuO transitions [68,81]. Figure 3a shows that the addition of H_2_O_2_ to the buffer solution affects the peak current values. The value of the maximum current for all peaks increases with increasing concentration of added peroxide (from 0 to 5 mM).

The mechanism of electron transfer in the modified electrode can be explained as follows: In this catalytic process, during the reduction of H_2_O_2_ on the CuO surface, Cu^2+^ is electrochemically reduced to Cu^+^ and H_2_O_2_ to O_2_. Then, Cu^+^ on the electrode surface is electrooxidized back to Cu^2+^, and the catalytic cycle is repeated [55,81–82].

Figure 3b shows CV curves for a pure Cu wire and CuO film obtained by copper annealing compared to a nanostructured CuO film obtained by chemical hydrothermal oxidation. All measurements were carried out in 0.1 M NaOH with the addition of 5 mM H_2_O_2_. The baseline shows the CV results for a buffer solution with no peroxide added. It can be seen that under identical measurement conditions the electrochemical response of the hydrothermally obtained film is significantly higher than the response from the thermally oxidised film, which indicates a significant contribution of the electrode nanostructuring process to an increase in the sensitivity of the sensor. This can be explained by the fact that petal-like CuO nanostructures provide a much larger surface area, with an increased number of active bonds and high-speed paths for analyte molecule transfer due to the high porosity of the surface, as well as more efficient mass diffusion and electron transfer processes compared to the less developed film. The sensitivity of pure CuO wire is significantly inferior to samples containing CuO.

Figure 3c,d displays the CV curves obtained at various pH values of buffer solution and various scanning speeds. It can be seen that the parameters pH 12.7 and *v* = 100 mV/s provide the result with maximum sensitivity. Figure 3e displays the electrode stability over multiple CV cycles. It can be seen that starting from the second scanning cycle the curve takes its characteristic shape. The value of the current peak changes slightly with time, which indicates that the electrode stabilizes after a short time. Small differences in the initial scan cycles may be due to the wetting of nanostructures.

In Figure 3f, the EIS curve and the corresponding equivalent circuit are presented. The absence of characteristic semicircles formed by RCs by the circuit elements indicates a low charge transfer resistance and the predominance of Warburg diffusion over other processes in the electrochemical system. Figure 3f shows an unambiguous change in the EIS curves as a reaction to the addition of small concentrations of H_2_O_2_ to the solution.

The active surface area of an electrode can be calculated using the Randles–Sevcik equation [83–85], which at 25 °C is:


[4] $$I_{\text{p}}=\left(2.69\times 10^{5}\right)n^{3/2}A\cdot C^{*}\cdot D^{1/2}\cdot v^{1/2},$$

where *I*_p_ represents the redox peak current (A), *n* is the number of electrons transferred in the redox reaction, *D* is the diffusion coefficient in solution (*D* = 6.8 × 10^−5^ cm^2^·s^−1^), C* is the concentration (mol·cm^−3^); *v* is the scan rate (100 mV·s^−1^), and *A* denotes the effective surface area of the electrode (cm^2^). The electrochemically active surface area was calculated to be 6.5 cm^2^, that is, five times larger than the geometrical surface area of a bare electrode, which indicates the presence of a well-developed nanostructured surface.

**Figure 4:**
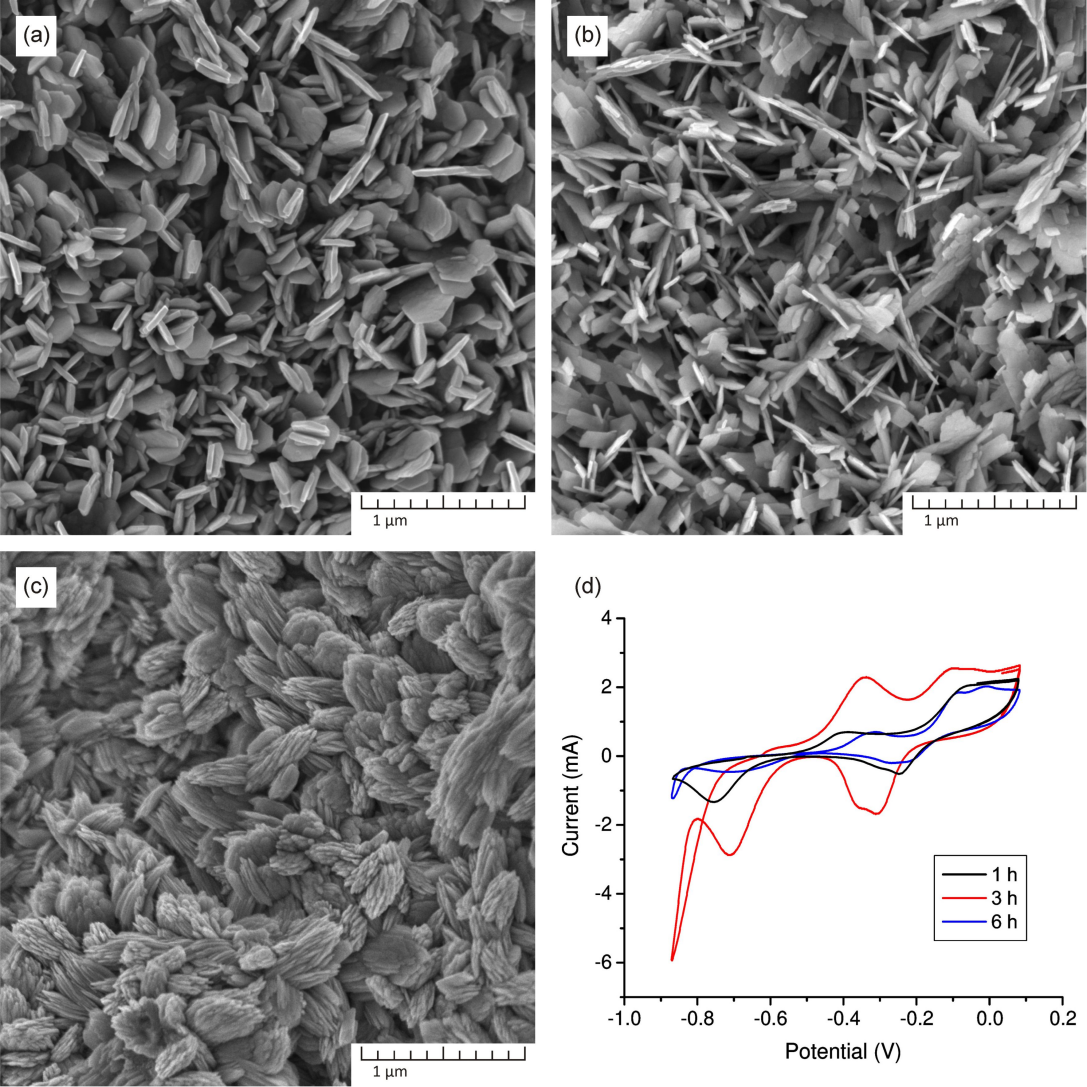
SEM images of CuO nanostructures obtained via hydrothermal oxidation method after (a) 1 h, (b) 3 h, and (c) 6 h. (d) CV curves of the CuO samples after 1, 3, and 6 h of synthesis time. Measurements were carried out in 0.1 M NaOH solution containing 1 mM H_2_O_2_.

Figure 4 displays the dependence of the sensor sensitivity on the morphology of CuO nanostructures obtained after different periods of synthesis time. It is shown that as a result of 1 h of growth, nanopetals are formed with a greater thickness and a significantly lower height than in the case of 3 h of growth. This change in aspect ratio leads to a decrease in the active surface area and, as a result, to a decrease in sensitivity (reduction of the current peak in the CV curves). An increase in the duration of hydrothermal synthesis to 6 h also leads to a change in the morphology of the nanostructures. The SEM picture shows that the nanoleaves grow together, forming dense spherical formations that are difficult for the solution to penetrate, which also leads to a decrease in the surface area and a deterioration in sensitivity (decrease of current peak value). Hence, it can be concluded that the chosen synthesis time of 3 h is optimal and provides maximum sensitivity.

**Figure 5:**
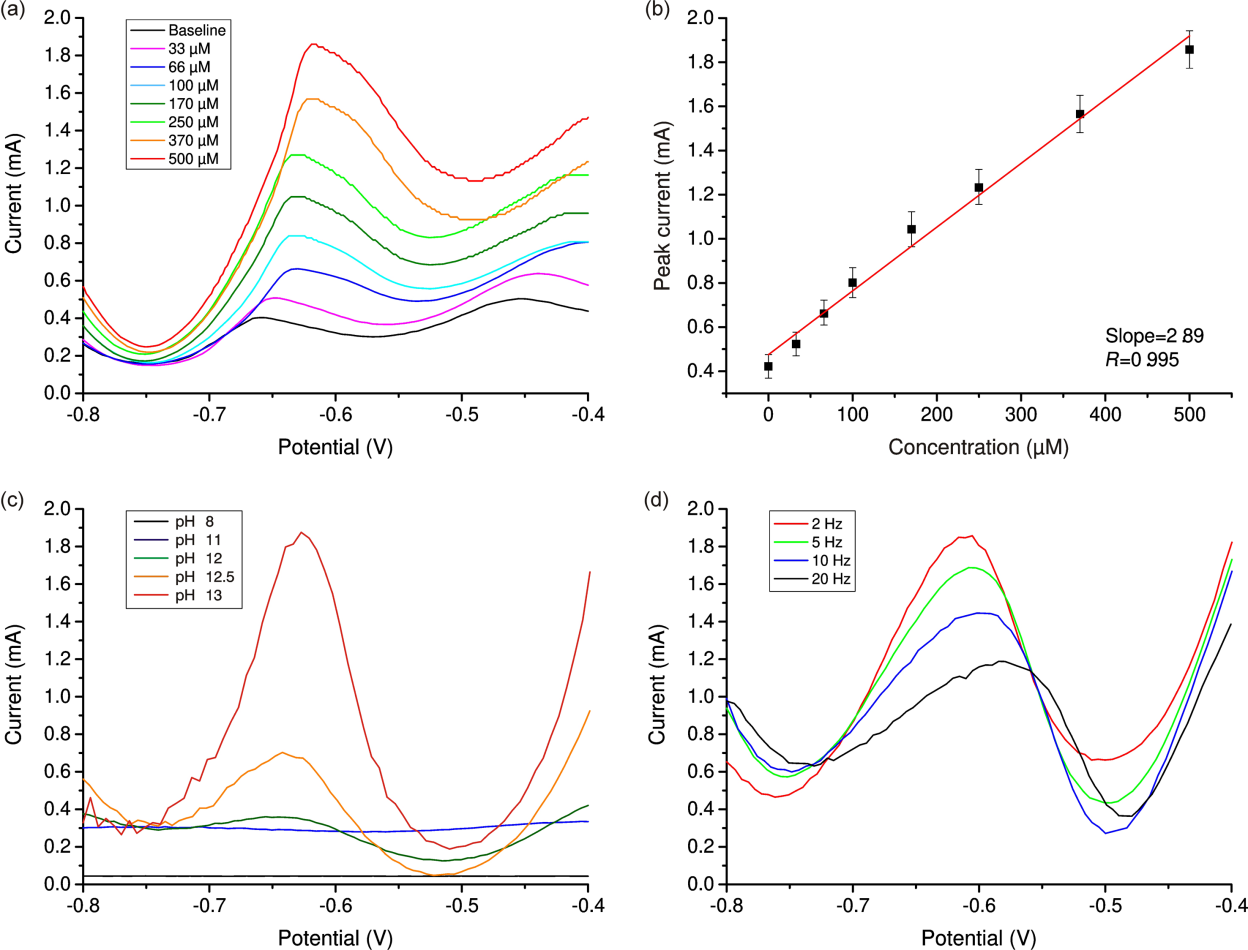
(a) DPV results for the nanostructured CuO electrode in 0.1 M NaOH buffer solution containing 33–500 μmol H_2_O_2_. (b) Dependence of the amperometric response on the concentration of added peroxide (SD = 3.5%, *n* = 5). (c) Comparison of DPV curves obtained at different pH values of buffer solution containing 500 µM H_2_O_2_. (d) Comparison of DPV curves obtained at different pulse frequences. Measurements were carried out in 0.1 M NaOH solution containing 500 µM H_2_O_2_.

Figure 5 shows the DPV results for the nanostructured CuO electrode. The measurements were carried out in 0.1 M NaOH buffer solution containing H_2_O_2_ at a concentration of 0–500 µM. The lowest considered concentration (33 μM) provides a noticeable electrochemical response, which indicates that the nanostructured CuO electrode has sufficiently high sensitivity. Figure 5b shows the dependence of the peak current values on the concentration of H_2_O_2_. The resulting dependence is linear over the entire concentration range.

Figure 5c,d displays the DPV curves at different pH values of buffer solution and different pulse frequency. It can be seen that the parameters pH 13 and 2 Hz provide the result with maximum sensitivity.

**Figure 6:**
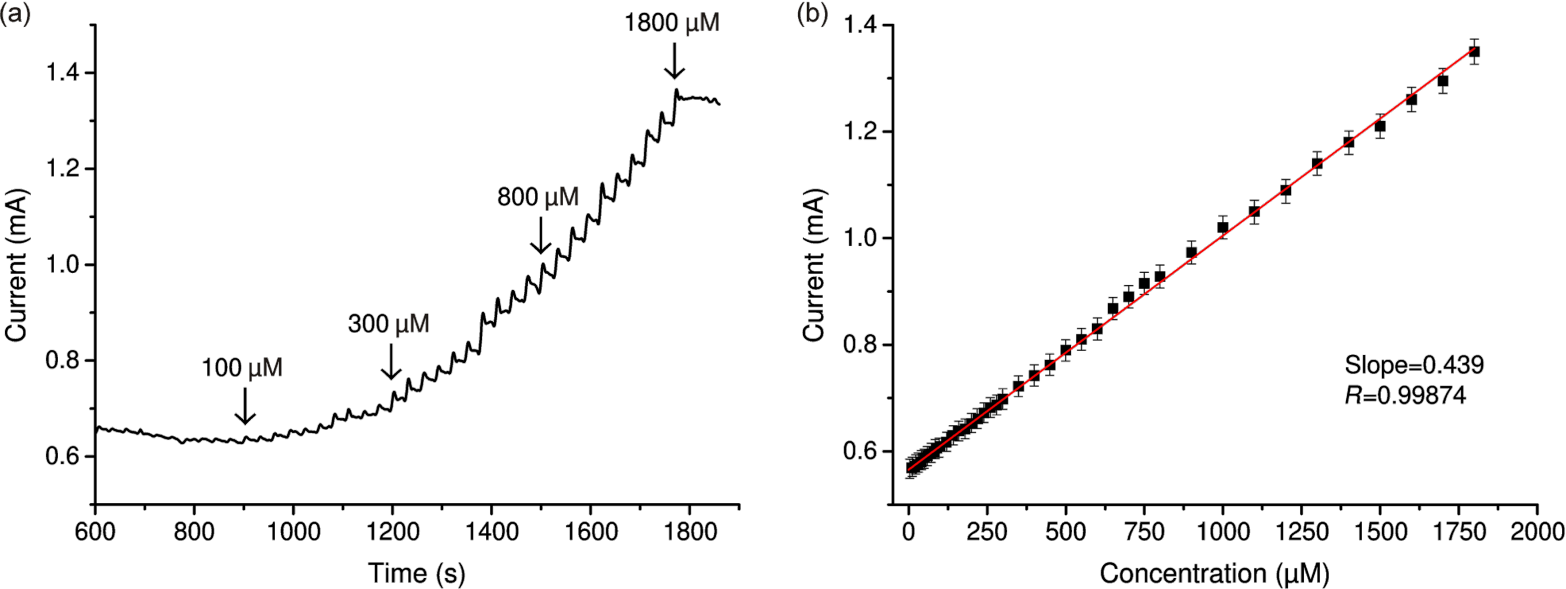
(a) Amperometric response of the nanostructured CuO electrode in 0.1 M NaOH with stepwise addition of H_2_O_2_ at concentrations from 10 to 1800 μM and (b) the corresponding calibration curve (SD = 3.5%, *n* = 5).

**Figure 7:**
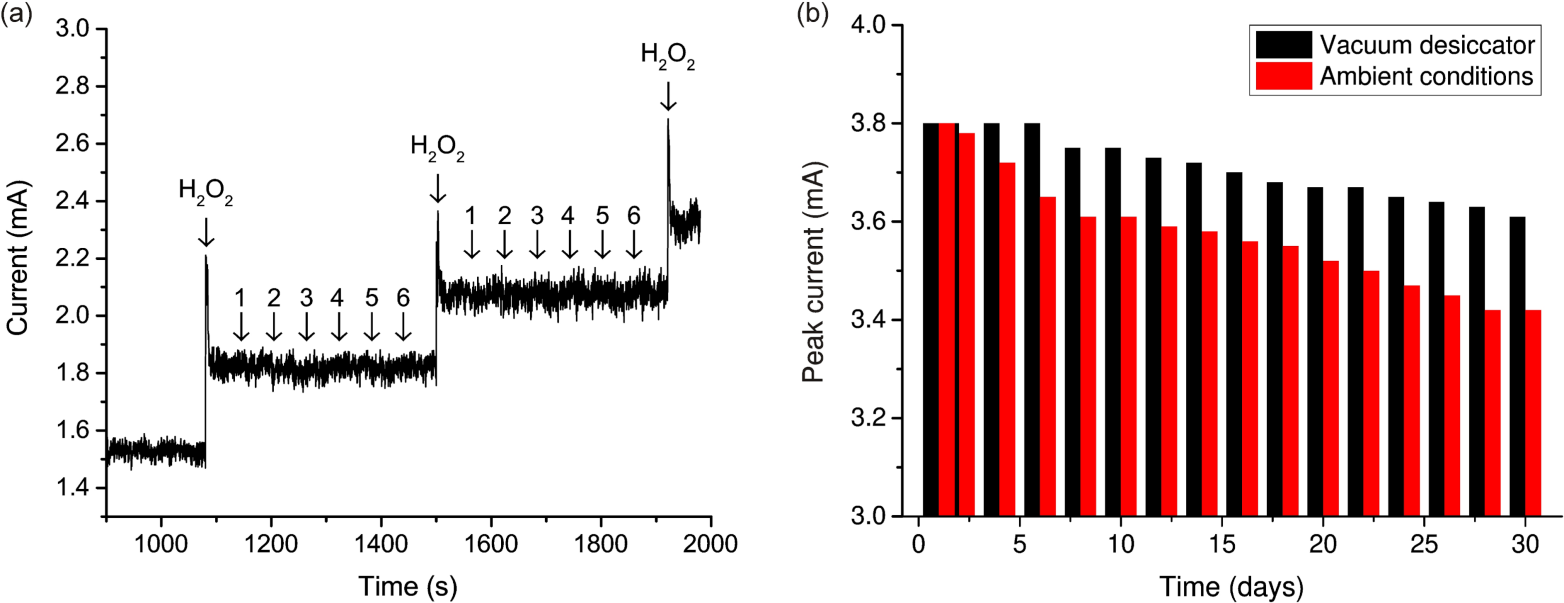
(a) Amperometric response of the nanostructured CuO electrode in 0.1 M NaOH with stepwise addition of H_2_O_2_ at concentrations from 100 to 300 μM and the most common interfering substances: (1) ascorbic acid, (2) uric acid, (3) dopamine, (4) NaCl, (5) glucose, and (6) acetaminophen. (b) Stability study and the dependence of the change in the electrochemical signal on the storage time of the samples.

Figure 6a and Figure 7a show typical curves of the amperometric response for nanostructured CuO electrodes. After H_2_O_2_ injection, a fast, stable, and sensitive amperometric response was observed. The sharp jump in the current when H_2_O_2_ is added can be explained by a local increase in the H_2_O_2_ concentration near the electrode. However, it can be seen that the current reaches a steady-state value after less than 5 s, and then does not change significantly before the next portion of H_2_O_2_ is added, forming a plateau (Figure 7). Figure 7b shows the calibration curve for the dependence of the catalytic current values on the concentration of H_2_O_2_.

A linear relationship was obtained in the range from 10 to 1800 µM (*R* = 0.99874). The sensitivity of the obtained CuO electrode is 439.19 μA·mM^−1^. The calculated limit of detection (LOD) is 1.34 μM, assuming signal-to-noise ratio of 3. The results indicate that the nanostructured CuO electrode can be used for accurate and precise detection of H_2_O_2_. The obtained results are comparable to several published studies where CuO nanostructures were used for electrode modification for H_2_O_2_ detection (Table 1).

**Table 1:** Analytical performance of the CuO sensor in this study compared with other reported H_2_O_2_ sensors.

Electrode	Morphology of nanostructured CuO	Linear range (μM)	Sensitivity (μA/mM)	LOD (μM)	Reference

Cu_2_O/GCE	nanocubes	0.3–7.80	—	64.4	[70]
CuO/APGE	nanoparticles	5–1600	4.75	0.21	[68]
CuO/Cu foil	nanopetals	10–960	5030	2.1	[56]
CuO/GCE	nanograss	10–900	80.4	5.5	[82]
CuO/rGO	nanoparticles	0.05–532	57.6	0.0043	[69]
CuO/PAN	3D nanoflowers	0.5–125	—	0.12	[77]
CuO/CoO	3D nanoleaf	2–4000	6349	1.4	[52]
CuO/SiNWs	nanoparticles	10–13180	22.27	1.6	[67]
CuO/Cu wire	nanopetals	10–1800	439.19	1.34	this work

For the successful practical application as a sensor material, a high selectivity of the obtained coating is of importance. Therefore, the selectivity of the petal-like CuO electrode was evaluated using four different interfering substances, namely ascorbic acid, uric acid, dopamine, and NaCl. These substances are most commonly encountered in clinical and pharmaceutical applications together with H_2_O_2_. They are also oxidizing agents that can react with CuO during electrochemical tests, leading to a false increase in the current signal. The amperometric response after sequential injection of 0.1 mM H_2_O_2_ and 0.1 mM interfering substance is shown in Figure 7a. There is an insignificant reaction of the sensor to the above substances, the current intensity of which is commensurate with the noise level. Thus, it can be concluded that the CuO petal-like electrode shows good selectivity for the detection of H_2_O_2_.

Table 2 shows the result of an amperometric study of real milk and mouthwash samples. As the possible amount of H_2_O_2_ can be below the detection limit, the samples were spiked with different amounts of H_2_O_2_ above the detection threshold and a standard sample recovery test was performed. It can be seen that the electrode has a high recovery rate (over 95% for all cases) and a low relative standard deviation for three samples of each spiked concentration not exceeding 5.5%. The results indicate that this sensor can be successfully used to detect hydrogen peroxide in real samples.

**Table 2:** Results of determination of hydrogen peroxide in real samples.

Milk				Mouthwash			

Added (μM)	Found (μM)	Recovery (%)	RSD (%) (*n* = 3)	Added (μM)	Found (μM)	Recovery (%)	RSD (%) (*n* = 3)

0	—	—	—	0	—	—	—
10	9.59	95.9	5.5	10	9.51	95.1	5.5
25	23.88	95.52	5.3	25	23.91	95.6	5.1
50	47.53	95.06	4.8	50	48.01	96.01	5.2
100	97.73	97.73	5.1	100	98.25	98.25	5.4

To assess the long-term stability of the sensor, the obtained samples were stored under ambient conditions for one and four weeks. Measurements were taken every second day. The stabilities of each sample were assessed by the degree of reduction of the current peak value in the CV curve. For samples stored under environmental conditions (20 °C, 40% relative humidity) for one week, the signal level remained at 95% of the initial value. For samples stored under environmental conditions for a month, the signal level remained at 90% of the initial value. The influence of the environment and degree of sample degradation can be significantly reduced by ensuring that samples are stored in a vacuum desiccator. After a week of desiccator storage, the samples had not lost their original electrochemical properties at all, and after a month of storage they retained 95% of their initial values (Figure 7b). After a month of storage, no significant morphological changes were observed, which proves the stability of the samples. These results show that the nanostructured CuO coating has long-term stability and resistance to environmental influences, which is another advantage compared to enzyme sensors.

## Conclusion

This article describes the preparation of a nanostructured coating of CuO and its application as a working electrode for the electrochemical determination of H_2_O_2_. The resulting coating is distinguished by high homogeneity and adhesion to the copper wire, which ensures high mechanical and chemical resistance of the sample. The nanostructured CuO coating develops a petal-shaped surface, which possesses significant peroxidase-like electrocatalytic activity, and makes it possible to detect H_2_O_2_ with a high degree of sensitivity compared to samples with less developed surface. It has been shown that the optimal time for hydrothermal synthesis is 3 h, since this period of time allows one to obtain a morphology with maximum electrochemical response towards H_2_O_2_.

The resulting electrode displays a linear current response in a concentration range from 10 to 1800 µM. The sensitivity of the resulting electrode was 439.19 μA·mM^−1^ and the calculated limit of detection (LOD) was 1.34 μM. The electrochemically active surface area was calculated to be 6.5 cm^2^. Sensitivity testing showed a lack of electrochemical response to the most common interfering substances, showing the high selectivity of this electrode. This study also showed high long-term stability of the resulting coating stored under ambient conditions (the signal level remained at 95% of the initial value after one week and at 90% after a month). Storage in a vacuum desiccator helps to improve the stability of samples (the signal level remained at 100% of the initial value after one week and at 95% after a month). Real milk sample and mouthwash analysis demonstrated a high recovery rate (over 95%), which makes this sensor suitable for qualitative and quantitative detection of H_2_O_2_.

Further research will be aimed at studying this sensor in healthcare to analyse changes in the concentration of H_2_O_2_ in biological fluids. Also, a promising option to study more complex analytes and to significantly increase the sensitivity is the use of this nanostructured CuO sensor as part of a multisensor system based on several types of metal oxides (e.g., Co_2_O_3_, TiO_2_, NiO, and Fe_2_O_3_).
